# Visible light sensitizer-catalyzed highly selective photo oxidation from thioethers into sulfoxides under aerobic condition

**DOI:** 10.1038/s41598-018-20631-7

**Published:** 2018-02-02

**Authors:** Cong Ye, Yanbin Zhang, Aishun Ding, Yong Hu, Hao Guo

**Affiliations:** 1 Department of Chemistry, Fudan University, 220 Handan Road, Shanghai, 200433 People’s Republic of China; 2 Department of Neonatology, Shanghai Children’s Hospital, Shanghai Jiao Tong University, Shanghai, 200040 People’s Republic of China

## Abstract

We report herein a visible light sensitizer-catalyzed aerobic oxidation of thioethers, affording sulfoxides in good to excellent yields. The loading of the catalyst was as low as 0.1 mol%. The selectivity was excellent. Mechanism studies showed both singlet oxygen and superoxide radical anion were likely involved in this transformation.

## Introduction

Sulfoxides are important fragments in organic synthesis^1–4^ and biologically active molecules^5^, including commercialized medicines^6,7^ and antiseptics^8^. Oxidation of thioethers into sulfoxides was the most straightforward pathway for the synthesis of sulfoxides^9–11^. Several methods in this field were developed during the past decades, including hydrogen peroxide oxidation^12–14^, metal complexes-catalyzed oxidation^15–20^, organocatalytic oxidation^21^, photo oxidation^22–27^, etc. However, stoichiometric external organic or inorganic oxidants were generally required in those reactions. Thus, a large amount of environmentally unfavorable wastes were generated during the production of sulfoxides. Another issue of those methods were the low selectivity between sulfoxides and over-oxidized by-product sulfones in many cases^14^. Although some catalytic system showed high selectivity, but the catalyst was too expensive to practical applications^15,17^. With the consideration of “Green Chemistry”, an environmentally friendly, energy-saving, atom-economical, and highly selective oxidation from thioethers to sulfoxides is required.

**Figure 1:**
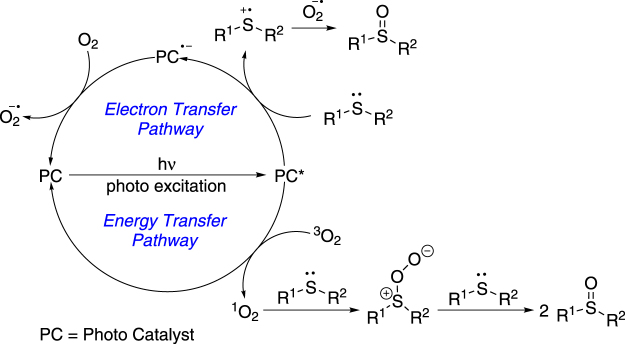
Photo oxidation of thioethers into sulfoxides.

Visible light has attracted wide attentions with its clean and abundant advantages. Outstanding works by MacMillan *et al*. showed the utilizations of visible light in organic reactions^28^. Two typical pathways normally proceeded in visible light catalysis: electron transfer and energy transfer. Ru or Ir complexes^29–33^ and some heteroatom-containing metal-free organic dyes^34–36^, which trend to grab or donate an electron in its excited state, are often used as the electron transfer catalyst. On the other hand, some rigid and conjugated organic compounds^37–39^, which can absorb visible light photon but are not capable of grabbing or donating electrons, could be used as energy transfer catalyst. Selective oxidations of thioethers into sulfoxides catalyzed by visible light photo catalysts have been widely studied^24–27^. Those reactions could also be classified to electron transfer process and energy transfer process as mentioned above (Fig. 1). In electron transfer process, superoxide ion (O_2_^−^) was the key oxidative intermediate^27^. Recently, Chao and Zhao reported a visible light-induced photo oxidation of thioethers using a dinuclear Ru-Cu complex as the catalyst^23^. While in energy transfer process, oxygen was directly excited to its singlet state (^1^O_2_) which served as the predominant oxidative species^25^. Notably, Vitamin B_2_ Derivative could achieve this reaction via both electron transfer process and energy transfer process^22^. Although visible light-induced selective oxidations of thioethers into sulfoxides under aerobic conditions have been reported, these reactions normally required expensive photo-catalysts with relatively high loading. Reactions with higher efficiency and lower cost were still required. Based on our continuous interest in photo oxidation reactions^40–42^, we decided to investigate whether thioxanthone derivatives would be an effective energy transfer catalyst in thioethers oxidation. Herein, we wish to report our recent results on visible light sensitizer-catalyzed aerobically selective oxidation of thioethers into sulfoxides.

## Results and Discussion

### Optimization and scope investigation

In the beginning, methyl phenyl thioether (**2a**) was chosen as the model substrate. The initial attempt was conducted under oxygen atmosphere at rt using 5 mol% of thioxanthone (**1a**) as the catalyst, toluene as the solvent and purple LED as the light source. A 7% NMR yield of methyl phenyl sulfoxide (**3a**) was formed with 55% of **2a** recovered (entry 1, Table 1). The low recovery was mainly caused by the loss during the process of rotary evaporation, since the boiling point of **2a** was low. Encouraged by the initial result, a screening of solvents was carried out. Only trace amount of **3a** was afforded when THF or CH_3_NO_2_ was used as the solvent (entries 2 and 3, Table 1). When cyclohexane, CH_2_Cl_2_, ethyl acetate (EA) or acetone was tested, the yield of **3a** was slightly increased (entries 4–7, Table 1). A dramatic improvement of the yield was observed using CH_3_CN as the solvent (entry 8, Table 1). But on the other hand, over oxidized product, methyl phenyl sulfone (**4a**), was also generated in a 4% NMR yield. The reaction in CH_3_OH gave an excellent yield of **3a** with increased selectivity of **3a**/**4a** (entry 9, Table 1). Thus, CH_3_OH was chosen as the best solvent. Next, modifications of thioxanthone derivatives were conducted aiming at promoting efficiency and selectivity. Thioxanthone derivatives were synthesized by the coupling of iodine compound with thiosalicylic acid followed by Friedel-Crafts reaction^43^. Reaction employing 2-chloro-thioxanthone (**1b**) showed better selectivity but lower yield (entry 10, Table 1). When 4-phenyl-thioxanthone (**1c**) was used as the catalyst, the reaction gave a 99% NMR yield of **3a** with the ratio of **3a**:**4a** being more than 99:1 (entry 11, Table 1). Then methoxy group was attached at 2-position of thioxanthone, but showed lower efficiency than **1c** (entry 12, Table 1). 1,4-Dihydroxy-thioxanthone (**1e**) was proved to be unfeasible in this transformation probably due to its low solubility (entry 13, Table 1). Thus, **1c** was chosen as the best catalyst. To our delight, decreasing the amount of **1c** till 0.1 mol% still gave excellent yield and selectivity (entries 14 and 15, Table 1). Further reducing the amount of **1c** to 0.01 mol% led to a sharply decreased yield (entry 16, Table 1). Finally, a series of control experiments were carried out indicating both catalyst and light was necessary for this reaction (entries 17 and 18, Table 1). Furthermore, considering the thermal effect caused by the purple LED light, the reaction was carried out at 50 °C without light. The result validated that no reaction took place at all even heated (entry 19, Table 1). Thus, Condition A (0.1 mol% of **1c**, CH_3_OH, purple LED, air atmosphere, and rt) was chosen as the optimized condition for further studies.

**Table 1:** Optimization of the reaction conditions^a^.


Entry	Solvent	Catalyst (%)	Time (h)	Yield (%)^b^			**3a**:**4a**
				**2a**	**3a**	**4a**	
1	toluene	**1a** (5)	4.5	55	7	0	−
2	THF	**1a** (5)	4.5	75	4	0	−
3	CH_3_NO_2_	**1a** (5)	4.5	25	2	0	−
4	cyclohexane	**1a** (5)	4.5	53	12	0	−
5	CH_2_Cl_2_	**1a** (5)	4.5	55	20	0	−
6	EA	**1a** (5)	4.5	36	22	0	−
7	acetone	**1a** (5)	4.5	34	41	0	−
8	CH_3_CN	**1a** (5)	4.5	0	83	4	95:1
9	CH_3_OH	**1a** (5)	4.5	0	95	3	97:1
10	CH_3_OH	**1b** (5)	5	0	93	1	99:1
11	CH_3_OH	**1c** (5)	5	0	99	<1	>99:1
12	CH_3_OH	**1d** (5)	5.5	0	94	1	99:1
13	CH_3_OH	**1e** (5)	4.5	22	3	0	−
14	CH_3_OH	**1c** (1)	5	0	99	1	99:1
15	CH_3_OH	**1c** (0.1)	5	0	99 (93)^c^	<1	>99:1
16	CH_3_OH	**1c** (0.01)	5	16	78	0	−
17	CH_3_OH	−	5	77	1	0	−
18^d^	CH_3_OH	**1c** (0.1)	5	78	0	0	−
19^d,e^	CH_3_OH	**1c** (0.1)	5	76	0	0	−

^a^All reactions were carried out using **2a** (1 mmol) and catalyst in solvent (5 mL) irradiated by a purple LED light at rt under air atmosphere^b^. The yield was determined by ^1^H NMR (400 MHz) analysis of the crude reaction mixture employing CH_2_Br_2_ (1 mmol) as the internal standard^c^. Isolated yield of **3a**^d^. The reaction was carried out without light^e^. The reaction was carried out at 50 °C.

**Figure 2:**
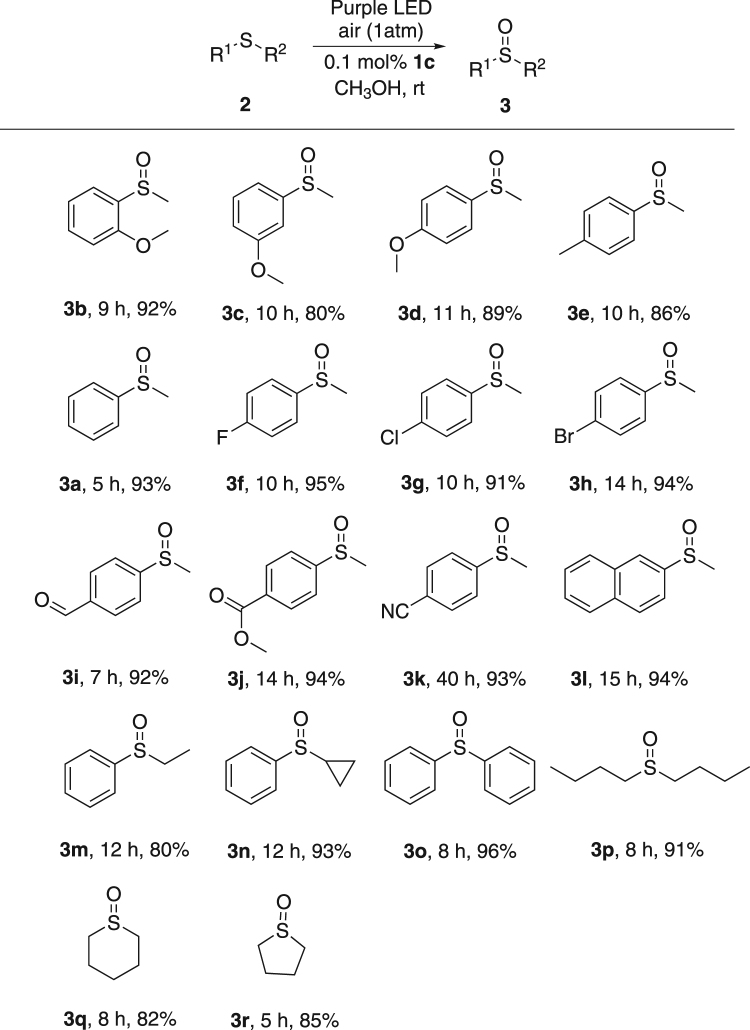
Photo oxidation under Condition A^a^. ^a^All reactions were carried out using **2** (1 mmol) and **1c** (0.1 mol%) in CH_3_OH (5 mL) irradiated by a purple LED light at rt under air atomsphere. Isolated yield was reported.

**Figure 3:**
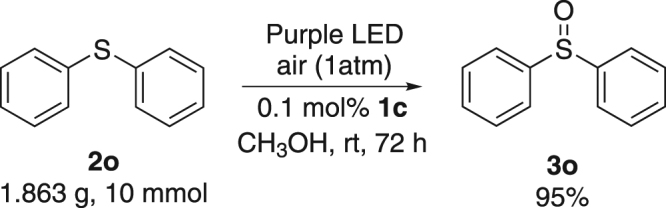
Gram scale synthesis of **3o**.

With the optimized reaction condition in hand, the scope of this oxidation was examined carefully. Some typical results are summarized in Fig. 2. Firstly, the electron effect of the aryl group in methyl aryl thioether was studied (**3a**–**k**). Excellent to good yields were obtained for methyl *o*-, *m*- or *p*-methoxyphenyl thioether (**3b**–**d**). Methyl 4-methylphenyl sulfoxide (**3e**) was formed in good yield from the corresponding reactant. In cases of substrates with halogen atom, excellent yields were obtained (**3f**–**h**). Substrates with strong electron withdrawing groups, like formyl (**3i**), methoxy carbonyl (**3j**), and nitrile (**3k**), were also tolerant in this reaction. When naphthyl ring was used instead of phenyl ring, a 94% isolated yield of methyl 2-naphthyl sulfoxide (**3l**) was generated. Secondly, we focused on the influence of alkyl group. Ethyl (**3m**) or cyclopropyl (**3n**) were applied instead of methyl. The corresponding yields were nice. Thirdly, diaryl thioether was also tolerant in this reaction, giving the corresponding sulfoxide (**3o**) in excellent yield and selectivity. Finally, aliphatic thioethers were examined. Di-*n*-butyl thioether led to an excellent yield of **3p**, while tetrahydro-2*H*-thiopyran and tetrahydrothiophene resulted in slightly lower yields of **3q** and **3r**, respectively. Scale-up reaction was also conducted using **2o** (Fig. 3) under Condition A. 95% of **3o** was afforded. This result showed the potential in organic synthesis.

### Mechanism studies

**Figure 4:**
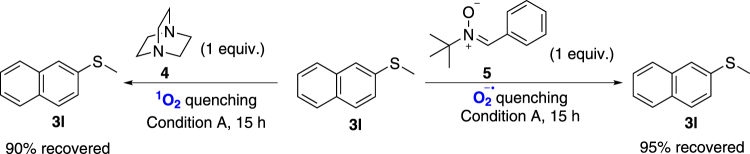
Quenching experiments.

To gain insight into the reaction mechanism, singlet oxygen quencher 1,4-diazabicyclo[2.2.2]octane (**4**)^44^ and superoxide radical anion quencher *N-tert*-Butyl-1-phenylmethanimine oxide (**5**)^45,46^ were added into the reaction system (Fig. 4), respectively. Severe inhibitions were observed in both cases. These results clearly indicated that both singlet oxygen and superoxide radical anion were likely involved in this transformation.

**Figure 5:**
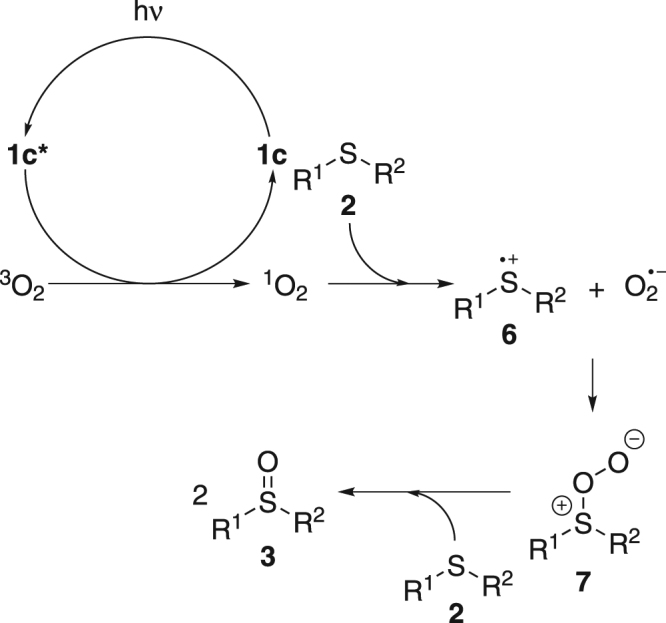
Proposed mechanism.

Based on the experiment results above and literature precedents^24,26,27^, a possible mechanism was proposed as shown in Fig. 5. 1c was excited upon the visible light irradiation and then sensitized oxygen to its singlet state^24^ which is more oxidative than normal triplet oxygen. Singlet oxygen could grab one electron from the lone pair electron of thioether **2**, forming thioether radical cation **6** and superoxide radical anion^27^. Then **6** could react with superoxide radical anion to give intermediate **7**^27^. **7** and another molecule of **2** further furnished **3** as the final product^27^.

## Conclusions

In conclusion, we developed a visible light sensitizer-catalyzed highly selective oxidation from thioethers into sulfoxides under aerobic condition. This reaction employed visible light as limitless energy source and 4-phenyl-9*H*-thioxanthen-9-one (**1c**) as metal-free catalyst with the loading as low as 0.1 mol%. This reaction showed high efficiency and selectivity with broad functional group tolerance. Gram-scale reaction could also be achieved under optimized conditions in nice yield and excellent selectivity. Mechanism studies indicated that both singlet oxygen and superoxide radical anion were likely involved in this transformation *via* energy transfer between visible light sensitizer and oxygen. Further applications of this reaction are in progress in our group.

## Methods

### Synthesis of methyl phenyl sulfoxide (3a)

A solution of **1c** (10 mg, 0.03 mmol) in CH_3_OH (100 mL) was prepared prior to use. **2a** (124 mg, 1.0 mmol), **1c** (3 mL, 0.1 mg/mL, 0.001 mmol), and CH_3_OH (2 mL) were added to a schlenk bottle which was equipped with a magnetic stirrer. The mixture was irradiated by a purple LED at rt under air atmosphere. The photoreaction was completed after 5 hours as monitored by TLC (eluent: petroleum ether/ethyl acetate = 10/1). The solvent was removed and the residue was purified by flash column chromatography on silica gel (eluent: petroleum ether→petroleum ether/ethyl acetate = 20/1→10/1→1/1) to afford **3a**^18^ as a solid (130 mg, 93%); ^1^H NMR (400 MHz, CDCl_3_) δ 7.68–7.63 (m, 2 H), 7.57–7.47 (m, 3 H), 2.72 (s, 3 H).

## Electronic supplementary material

Supporting Information
