# Supplementary material for: Visible light sensitizer-catalyzed highly selective photo oxidation from thioethers into sulfoxides under aerobic condition
Source: Sci Rep. 2018 Feb 2;8:2205. doi: 10.1038/s41598-018-20631-7 (PMC5797238; doi:10.1038/s41598-018-20631-7)
Supplement: Supplementary file 1 — Supporting Information [file 41598_2018_20631_MOESM1_ESM.pdf]

Supplementary Information

Visible light sensitizer-catalyzed highly selective photo oxidation from thioethers into sulfoxides under aerobic condition

Cong Ye,[1] *Yanbin Zhang*,[1] *Aishun Ding*,[1] *Yong Hu*,[2,*] *Hao Guo*[1,*]

[1] Department of Chemistry, Fudan University, 220 Handan Road, Shanghai 200433, People's Republic of China

[2] Department of Neonatology, Shanghai Children's Hospital, Shanghai Jiao Tong University, Shanghai, 200040, People's Republic of China

* To whom correspondence should be addressed, E-mail: Hao_Guo@fudan.edu.cn (Tel: +86-21-55664361, Fax: +86-21-55664361); huyongcn@163.com (Tel: +86-21-62474880).

Experimental Section	S2
NMR Spectra	S10
References	S20

Experimental Section

General experimental methods

All the photo reactions were carried out using purple LED (1 m strip × 2, Greethink 5050, 12 V/m) at a distance of 8-10 cm at rt under air atmosphere unless stated otherwise. ^{1}H (400 MHz), and ^{13}C (100 MHz) NMR spectra of samples in $CDCl_3$ were recorded on an AVANCE III 400 spectrometer. Melting points were determined on a WRS-2 apparatus. IR spectra were recorded on an Avatar 360 FT-IR spectrometer. HRMS (ESI) determinations were carried out on a Bruker Daltonics micrOTOF II spectrometer. Compounds **1a-b**, **2a-i** and **2k-r** were commercial available. Compounds **1d**,[1] **1e**[2] and **2j**[3] were prepared according to literature procedures.

Synthesis of 4-phenyl-9*H*-thioxanthen-9-one (1c)[1]

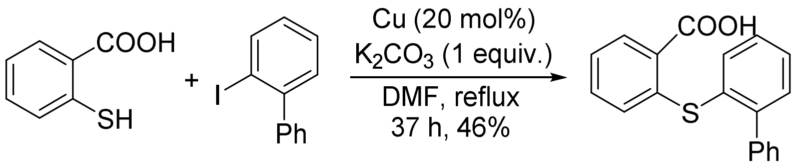

To a solution of thiosalicylic acid (3.095 g, 20.1 mmol) in DMF (60 mL) was added 2-iodo-1,1'-biphenyl (3.5 mL, 19.9 mol), K_2CO_3 (2.765 g, 20.0 mmol) and Cu powder (260 mg, 4.1 mmol). Then the mixture was warmed to reflux. After 37 h, the mixture was cooled to rt and filtered. The residue was dried under high vacuum, affording 2-([1,1'-biphenyl]-2-ylthio)benzoic acid (2.820 g, 46%) as the crude product and used without further purification.

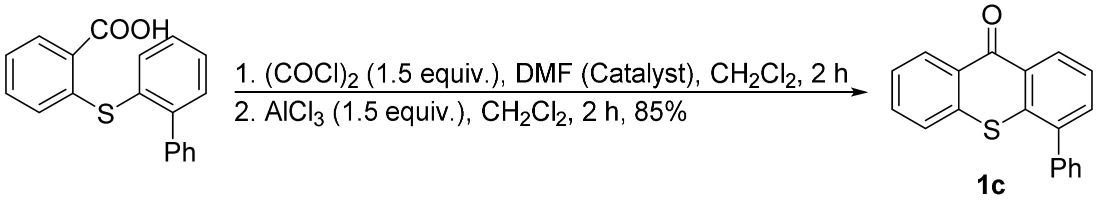

Crude 2-([1,1'-biphenyl]-2-ylthio)benzoic acid (3.065 g, 10.0 mmol) was placed in a dry 100 mL three-necked flask under a positive pressure of argon. Then dry CH_2Cl_2 (30 mL) were added and the reaction mixture was cooled to 0 °C. A few drops of DMF were added followed by the addition of oxalyl chloride (1.3 mL, 16.3 mmol) with stirring. The reaction was stirred until the suspension had completely dissolved. The product was concentrated in vacuum and dried under high vacuum to give the acid

chloride. Then under a positive pressure of argon, dry CH_2Cl_2 (30 mL) were added and the mixture was stirred at rt. $AlCl_3$ (2.012 g, 15.3 mmol) was added slowly and the reaction was stirred for 2 h. Then the reaction was quenched with H_2O and extracted with CH_2Cl_2. The combined organic phases were washed with saturated $NaHCO_3$ and dried over $MgSO_4$, filtered and concentrated in vacuum. The product was purified by flash chromatography on silica gel (eluent: petroleum ether/ethyl acetate = petroleum ether→20/1→10/1) to give **1c** as a solid (2.448 g, 85%); mp 202.3-202.6 °C (ethyl acetate/petroleum ether); ^{1}H NMR (400 MHz, $CDCl_3$) δ 8.72-8.64 (m, 1 H), 8.61-8.56 (m, 1 H), 7.58-7.40 (m, 10 H). ^{13}C NMR (100 MHz, $CDCl_3$) δ 180.3, 139.7, 138.5, 137.5, 136.6, 133.4, 132.2, 129.8, 129.6, 129.0, 128.6, 128.5, 126.2, 125.7; IR (neat) 1632, 1598, 1549, 1455, 1418 cm^{-1}; HRMS (ESI) calcd for $C_{19}H_{12}OS$ (M^+) 289.0682, found 289.0683.

Typical Procedure I for the photoreaction.

Synthesis of methyl phenyl sulfoxide (3a)[4]

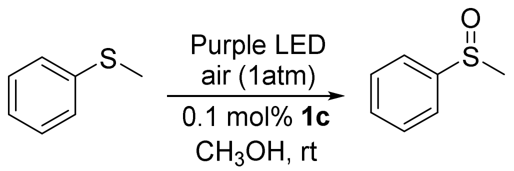

2a **3a**

A solution of **1c** (10 mg, 0.03 mmol) in CH_3OH (100 mL) was prepared prior to use. **2a** (124 mg, 1.0 mmol), **1c** (0.1 mg/mL, 3 mL, 0.001 mmol), and CH_3OH (2 mL) were added to a schlenk bottle which was equipped with a magnetic stirrer. The mixture was irradiated by a purple LED at rt under air atmosphere. The photoreaction was completed after 5 hours as monitored by TLC (eluent: petroleum ether/ethyl acetate = 10/1). The solvent was removed and the residue was purified by flash column chromatography on silica gel (eluent: petroleum ether → petroleum ether/ethyl acetate = 20/1→10/1→1/1) to afford **3a** as a solid (130 mg, 93%); ^{1}H NMR (400 MHz, $CDCl_3$) δ 7.68-7.63 (m, 2 H), 7.57-7.47 (m, 3 H), 2.72 (s, 3 H).

The following compounds were prepared according to Typical Procedure I.

(1) 2-Methoxyphenyl methyl sulfoxide (3b)[6]

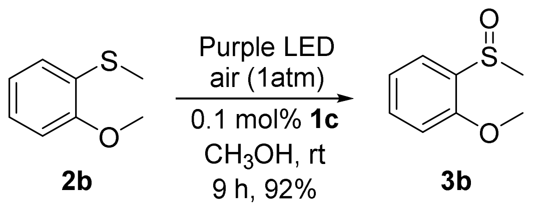

The reaction of **2b** (154 mg, 1.0 mmol), **1c** (0.1 mg/mL, 3 mL, 0.001 mmol), and CH_3OH (2 mL) afforded **3b** as a liquid (157 mg, 92%); ^1H NMR (400 MHz, $CDCl_3$) δ 7.81 (dd, J = 7.6, 1.6 Hz, 1 H), 7.47-7.42 (m, 1 H), 7.20-7.13 (m, 1 H), 6.93 (d, J = 8.4 Hz, 1 H), 3.88 (s, 3 H), 2.77 (s, 3 H).

(2) 3-Methoxyphenyl methyl sulfoxide (3c)[5]

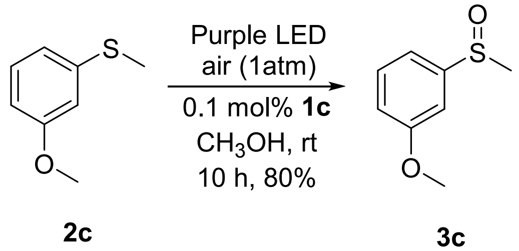

The reaction of **2c** (154 mg, 1.0 mmol), **1c** (0.1 mg/mL, 3 mL, 0.001 mmol), and CH_3OH (2 mL) afforded **3c** as a liquid (137 mg, 80%); ^1H NMR (400 MHz, $CDCl_3$) δ 7.42 (t, J = 8.0 Hz, 1 H), 7.26-7.25 (m, 1 H), 7.14 (d, J = 7.6 Hz, 1 H), 7.02 (dd, J = 8.4, 2.4 Hz, 1 H), 3.87 (s, 3 H), 2.73 (s, 3 H).

(3) 4-Methoxyphenyl methyl sulfoxide (3d)[4]

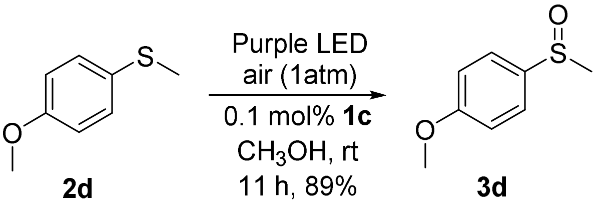

The reaction of **2d** (154 mg, 1.0 mmol), **1c** (0.1 mg/mL, 3 mL, 0.001 mmol), and CH_3OH (2 mL) afforded **3d** as a liquid (152 mg, 89%); ^1H NMR (400 MHz, $CDCl_3$) δ 7.60 (d, J = 8.8 Hz, 2 H), 7.03 (d, J = 8.8 Hz, 2 H), 3.85 (s, 3 H), 2.70 (s, 3 H).

(4) 4-Methylphenyl methyl sulfoxide (3e)[4]

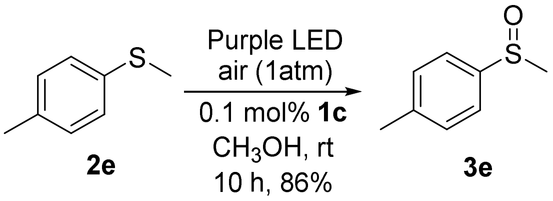

The reaction of **2e** (138 mg, 1.0 mmol), **1c** (0.1 mg/mL, 3 mL, 0.001 mmol), and CH_3OH (2 mL) afforded **3e** as a liquid (133 mg, 86%); ^{1}H NMR (400 MHz, $CDCl_3$) δ 7.54 (d, *J* = 8.0 Hz, 2 H), 7.33 (d, *J* = 8.0 Hz, 2 H), 2.71 (s, 3 H), 2.42 (s, 3 H).

(5) 4-Fluorophenyl methyl sulfoxide (3f)[4]

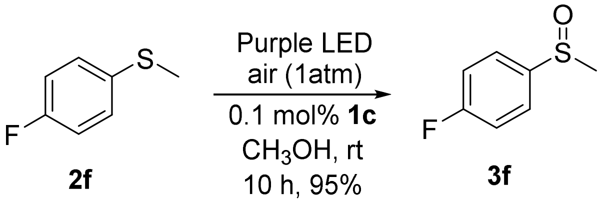

The reaction of **2f** (142 mg, 1.0 mmol), **1c** (0.1 mg/mL, 3 mL, 0.001 mmol), and CH_3OH (2 mL) afforded **3f** as a liquid (150 mg, 95%); ^{1}H NMR (400 MHz, $CDCl_3$) δ 7.71-7.62 (m, 2 H), 7.28-7.20 (m, 2 H), 2.73 (s, 3 H).

(6) 4-Chlorophenyl methyl sulfoxide (3g)[4]

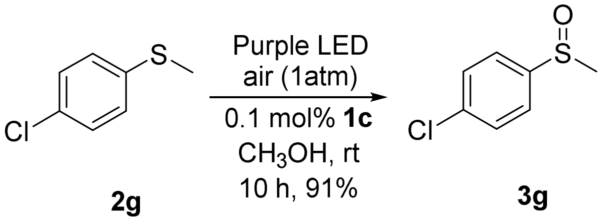

The reaction of **2g** (159 mg, 1.0 mmol), **1c** (0.1 mg/mL, 3 mL, 0.001 mmol), and CH_3OH (2 mL) afforded **2g** as a liquid (159 mg, 91%); ^{1}H NMR (400 MHz, $CDCl_3$) δ 7.61 (d, *J* = 8.6 Hz, 2 H), 7.51 (d, *J* = 8.6 Hz, 2 H), 2.73 (s, 3 H).

(7) 4-Bromophenyl methyl sulfoxide (3h)[4]

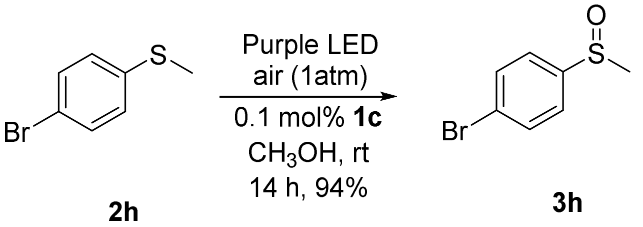

The reaction of **2h** (203 mg, 1.0 mmol), **1c** (0.1 mg/mL, 3 mL, 0.001 mmol), and CH_3OH (2 mL) afforded **3h** as a solid (205 mg, 94%); ^{1}H NMR (400 MHz, $CDCl_3$) δ

7.67 (d, J = 8.4 Hz, 2 H), 7.53 (d, J = 8.4 Hz, 2 H), 2.73 (s, 3 H).

(8) 4-Formylphenyl methyl sulfoxide (3i)[7]

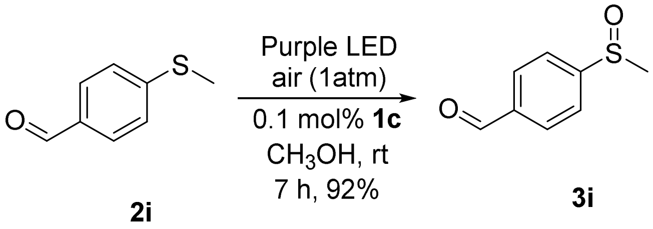

The reaction of **2i** (152 mg, 1.0 mmol), **1c** (0.1 mg/mL, 3 mL, 0.001 mmol), and CH_3OH (2 mL) afforded **3i** as a solid (155 mg, 92%); ^{1}H NMR (400 MHz, $CDCl_3$) δ 10.10 (s, 1 H), 8.06 (d, J = 8.0 Hz, 2 H), 7.84 (d, J = 8.0 Hz, 2 H), 2.80 (s, 3 H).

(9) 4-(Methoxycarbonyl)phenyl methyl sulfoxide (3j)[8]

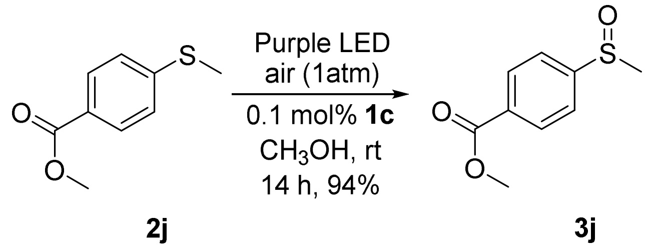

The reaction of **2j** (182 mg, 1.0 mmol), **1c** (0.1 mg/mL, 3 mL, 0.001 mmol), and CH_3OH (2 mL) afforded **3j** as a solid (187 mg, 94%); ^{1}H NMR (400 MHz, $CDCl_3$) δ 8.19 (d, J = 8.2 Hz, 2 H), 7.75 (d, J = 8.2 Hz, 2 H), 3.96 (s, 3 H), 2.79 (s, 3 H).

(10) 4-Cyanophenyl methyl sulfoxide (3k)[4]

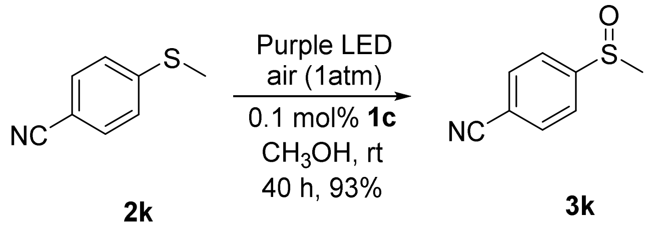

The reaction of **2k** (149 mg, 1.0 mmol), **1c** (0.1 mg/mL, 3 mL, 0.001 mmol), and CH_3OH (2 mL) afforded **3k** as a solid (154 mg, 93%); ^{1}H NMR (400 MHz, $CDCl_3$) δ 7.85 (d, J = 8.4 Hz, 2 H), 7.78 (d, J = 8.4 Hz, 2 H), 2.78 (s, 3 H).

(11) Methyl 2-naphthyl sulfoxide (3l)[5]

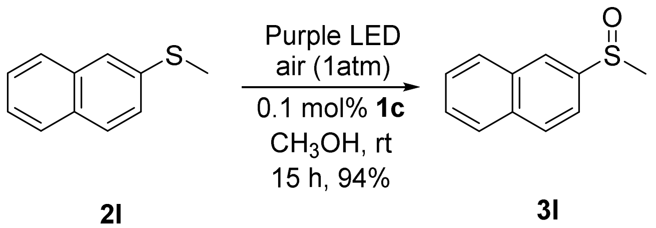

2l **3l**

The reaction of **2l** (174 mg, 1.0 mmol), **1c** (0.1 mg/mL, 3 mL, 0.001 mmol), and CH_3OH (2 mL) afforded **3l** as a solid (179 mg, 94%); ^1H NMR (400 MHz, $CDCl_3$) δ 8.18 (s, 1 H), 7.95-7.85 (m, 3 H), 7.59-7.50 (m, 3 H), 2.74 (s, 3 H).

(12) Ethyl phenyl sulfoxide (3m)[4]

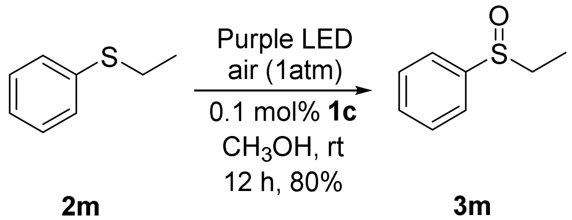

2m **3m**

The reaction of **2m** (138 mg, 1.0 mmol), **1c** (0.1 mg/mL, 3 mL, 0.001 mmol), and CH_3OH (2 mL) afforded **3m** as a liquid (123 mg, 80%); ^1H NMR (400 MHz, $CDCl_3$) δ 7.65-7.57 (m, 2 H), 7.55-7.46 (m, 3 H), 2.96-2.84 (m, 1 H), 2.82-2.70 (m, 1 H), 1.91 (t, J = 7.4 Hz, 3 H).

(13) Cyclopropyl phenyl sulfoxide (3n)[5]

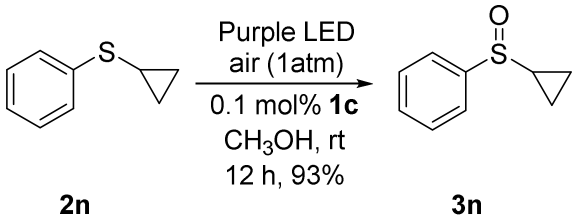

2n **3n**

The reaction of **2n** (150 mg, 1.0 mmol), **1c** (0.1 mg/mL, 3 mL, 0.001 mmol), and CH_3OH (2 mL) afforded **3n** as a liquid (156 mg, 93%); ^1H NMR (400 MHz, $CDCl_3$) δ 7.70-7.62 (m, 2 H), 7.55-7.44 (m, 3 H), 2.31-2.20 (m, 1 H), 1.25-1.15 (m, 1 H), 1.07-0.84 (m, 3 H).

(14) Diphenyl sulfoxide (3o)[4]

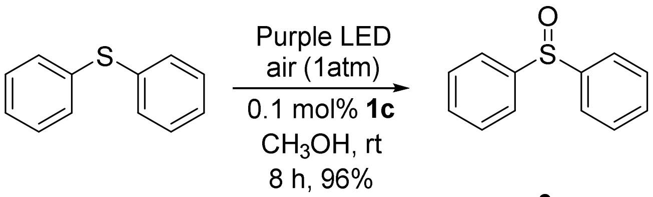

2o **3o**

The reaction of **2o** (186 mg, 1.0 mmol), **1c** (0.1 mg/mL, 3 mL, 0.001 mmol), and CH_3OH (2 mL) afforded **3o** as a solid (195 mg, 96%); ^1H NMR (400 MHz, $CDCl_3$) δ 7.69-7.60 (m, 4 H), 7.50-7.40 (m, 6 H).

(15) Dibutyl sulfoxide (3p)[6]

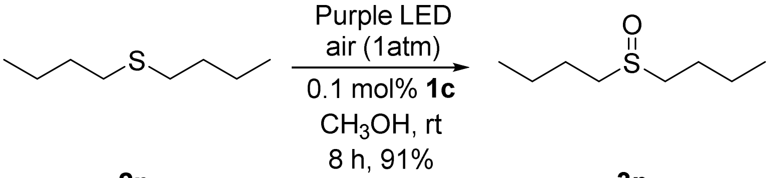

2p **3p**

The reaction of **2p** (146 mg, 1.0 mmol), **1c** (0.1 mg/mL, 3 mL, 0.001 mmol), and CH_3OH (2 mL) afforded **3p** as a liquid (147 mg, 91%); ^1H NMR (400 MHz, $CDCl_3$) δ 2.75-2.60 (m, 4 H), 1.81-1.69 (m, 4 H), 1.56-1.43 (m, 4 H), 0.97 (t, J = 7.4 Hz, 6 H).

(16) Thiane 1-oxide (3q)[9]

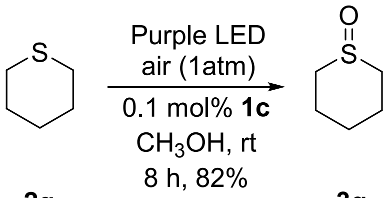

2q **3q**

The reaction of **2q** (102 mg, 1.0 mmol), **1c** (0.1 mg/mL, 3 mL, 0.001 mmol), and CH_3OH (2 mL) afforded **3q** as a solid (97 mg, 82%); ^1H NMR (400 MHz, $CDCl_3$) δ 2.95-2.88 (m, 2 H), 2.87-2.73 (m, 2 H), 2.27-2.20 (m, 2 H), 1.72-1.56 (m, 4 H).

(17) Tetramethylene sulfoxide (3r)[10]

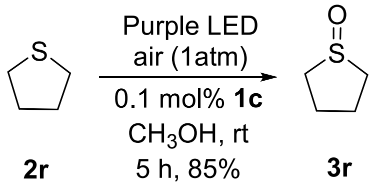

2r **3r**

The reaction of **2r** (88 mg, 1.0 mmol), **1c** (0.1 mg/mL, 3 mL, 0.001 mmol), and CH_3OH (2 mL) afforded **3r** as a liquid (89 mg, 85%); ^1H NMR (400 MHz, $CDCl_3$) δ 2.97-2.81 (m, 4 H), 2.49-2.41 (m, 2 H), 2.11-1.97 (m, 2 H).

(18) The gram-scale oxidation of 2o.

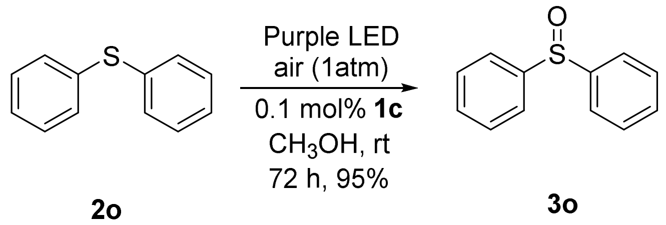

The reaction of **2o** (1.863 g, 10.0 mmol), **1c** (3 mg, 0.1 mmol), and CH_3OH (20 mL) afforded **3o** as a solid (1.912 g, 95%).

NMR Spectra

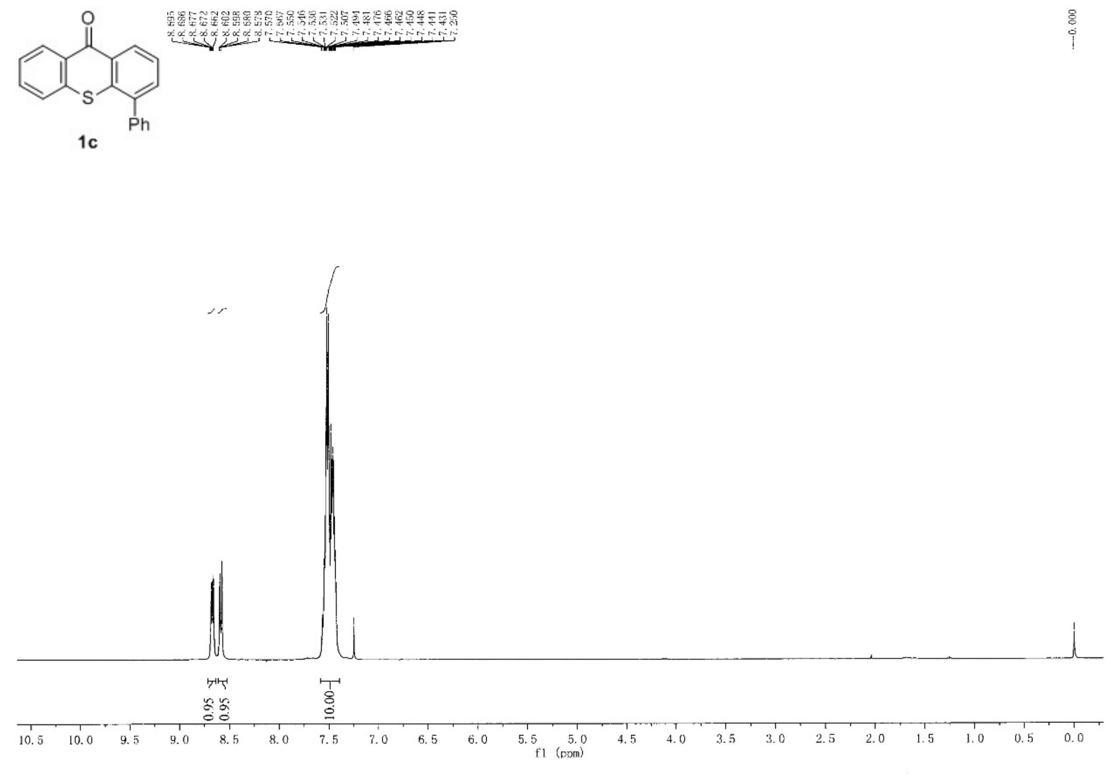

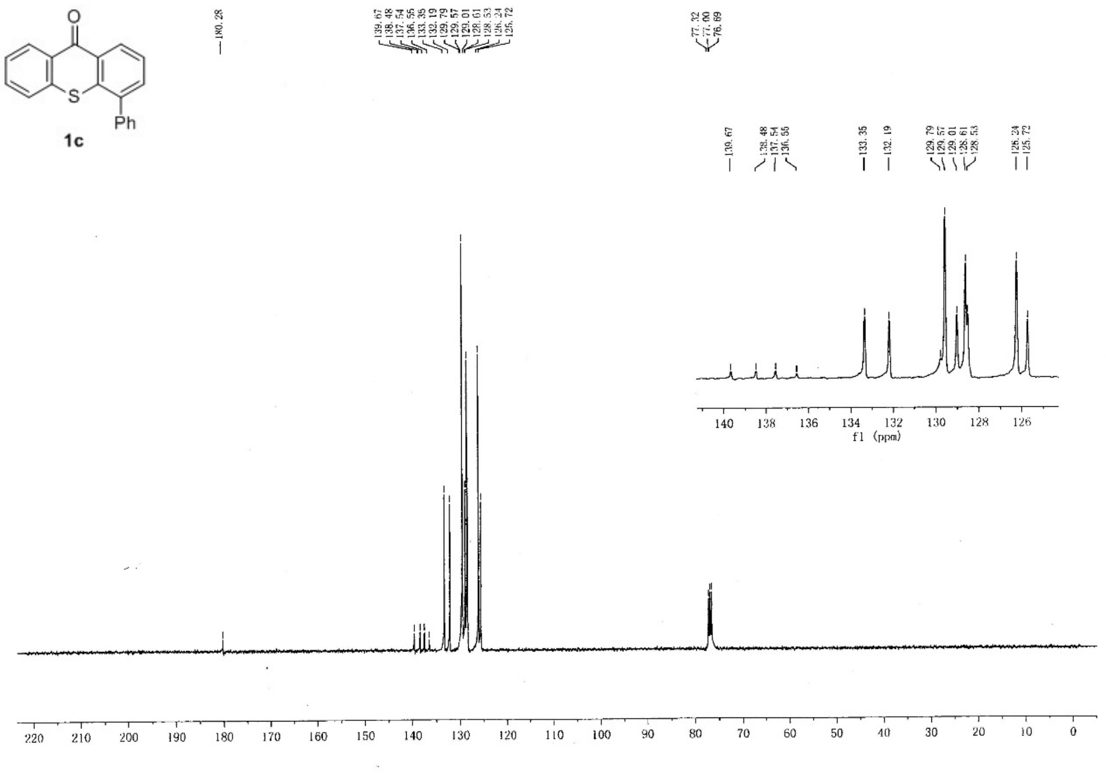

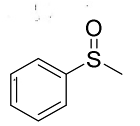

3a

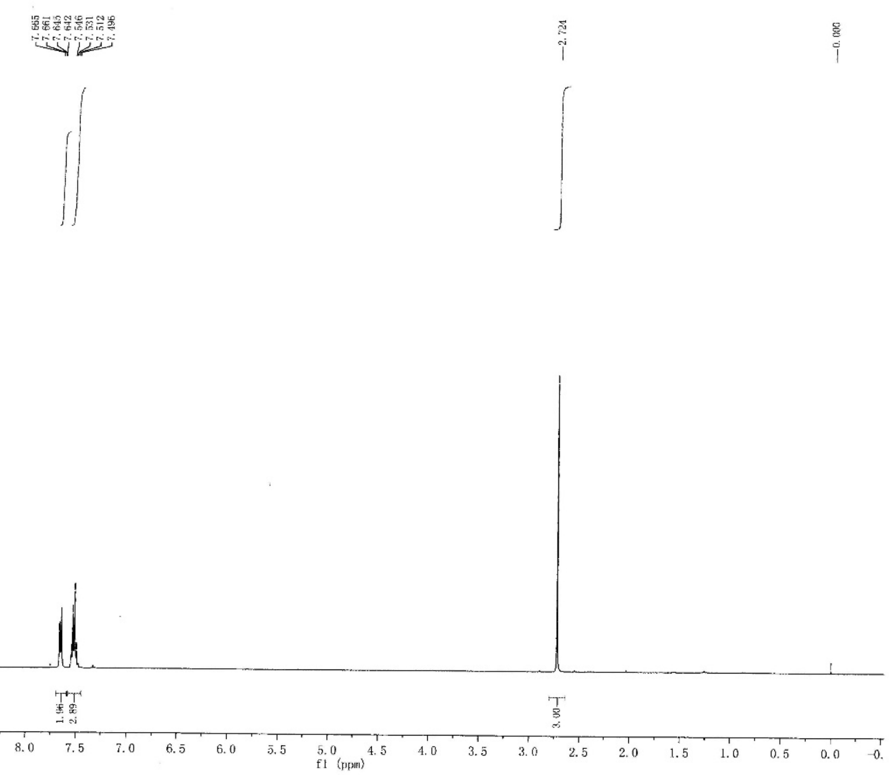
7.665
7.661
7.645
7.642
7.546
7.531
7.512
7.496
2.724
0.000
1.96
2.89
3.00
10.0 9.5 9.0 8.5 8.0 7.5 7.0 6.5 6.0 5.5 5.0 4.5 4.0 3.5 3.0 2.5 2.0 1.5 1.0 0.5 0.0
f1 (ppm)

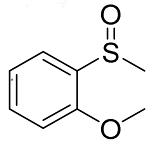

3b

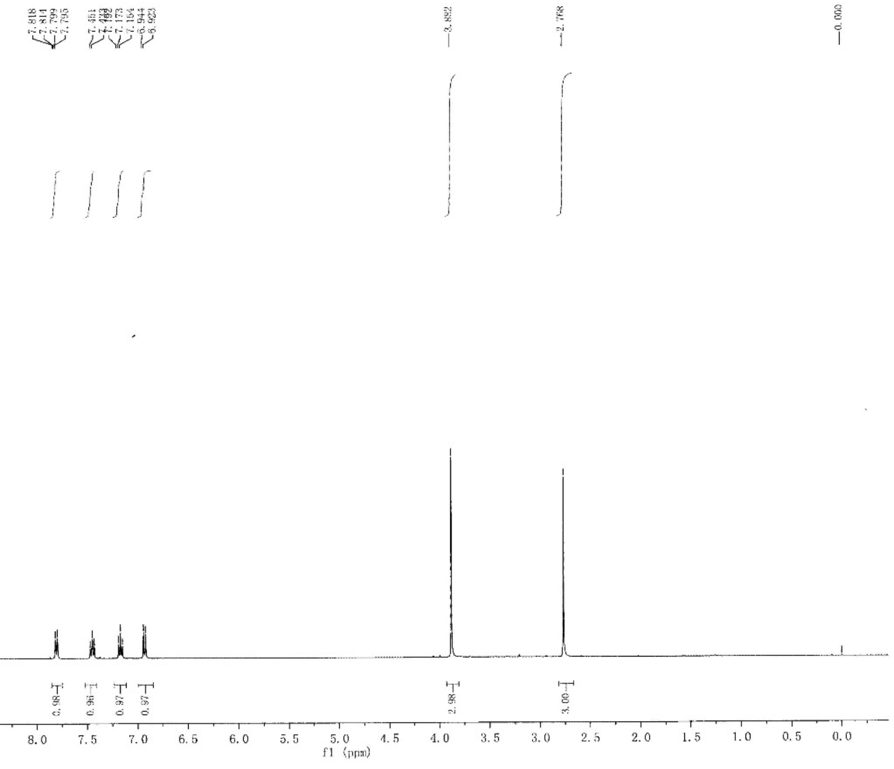
7.818
7.811
7.799
7.795
7.451
7.173
7.154
6.944
6.923
3.882
2.768
0.000
0.98
0.96
0.97
0.97
2.98
3.00
10.0 9.5 9.0 8.5 8.0 7.5 7.0 6.5 6.0 5.5 5.0 4.5 4.0 3.5 3.0 2.5 2.0 1.5 1.0 0.5 0.0
f1 (ppm)

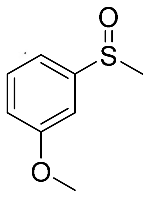

3c

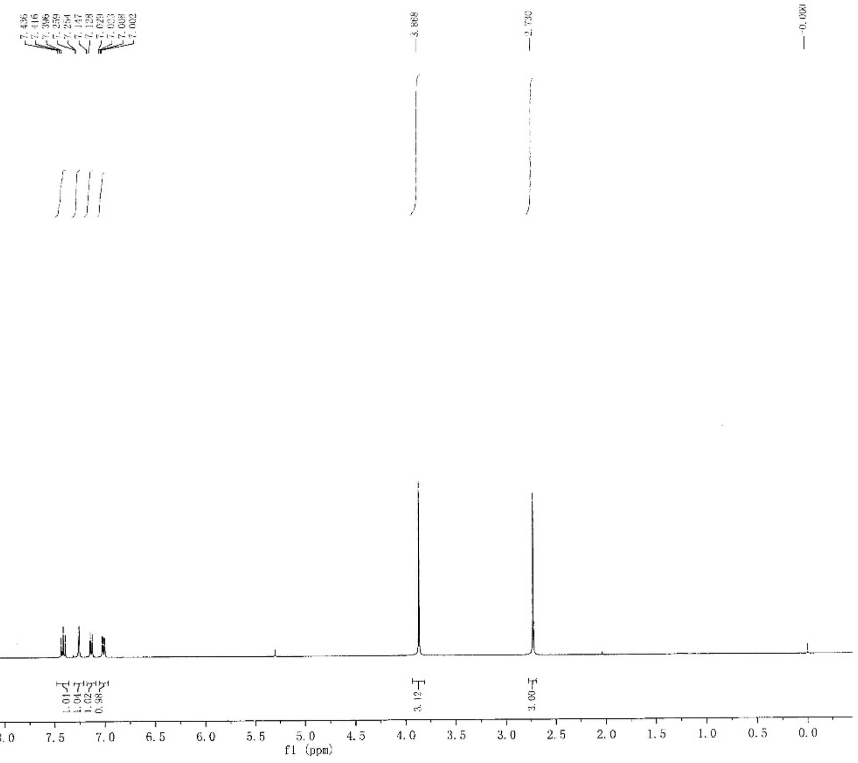
7.436
7.416
7.396
7.259
7.254
7.147
7.128
7.029
7.023
7.008
7.002
3.868
2.732
0.000
1.01
1.04
1.02
0.98
3.12
3.09
10.0 9.5 9.0 8.5 8.0 7.5 7.0 6.5 6.0 5.5 5.0 4.5 4.0 3.5 3.0 2.5 2.0 1.5 1.0 0.5 0.0
f1 (ppm)

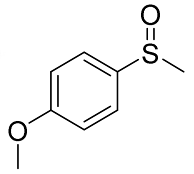

3d

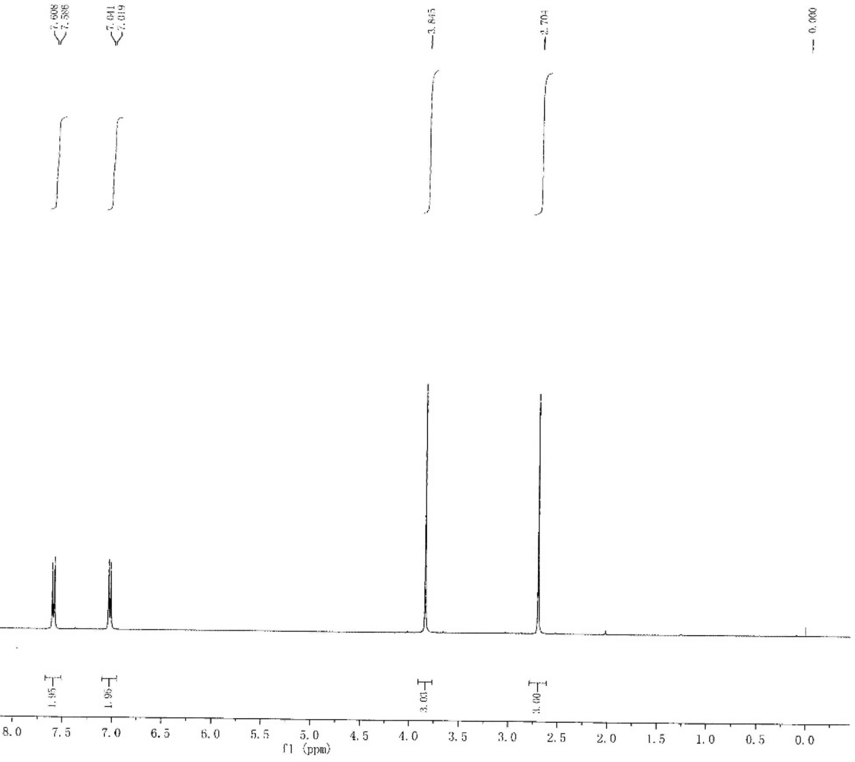
7.608
7.586
7.041
7.019
3.845
2.704
0.000
1.95
1.96
3.03
3.00
10.0 9.5 9.0 8.5 8.0 7.5 7.0 6.5 6.0 5.5 5.0 4.5 4.0 3.5 3.0 2.5 2.0 1.5 1.0 0.5 0.0
f1 (ppm)

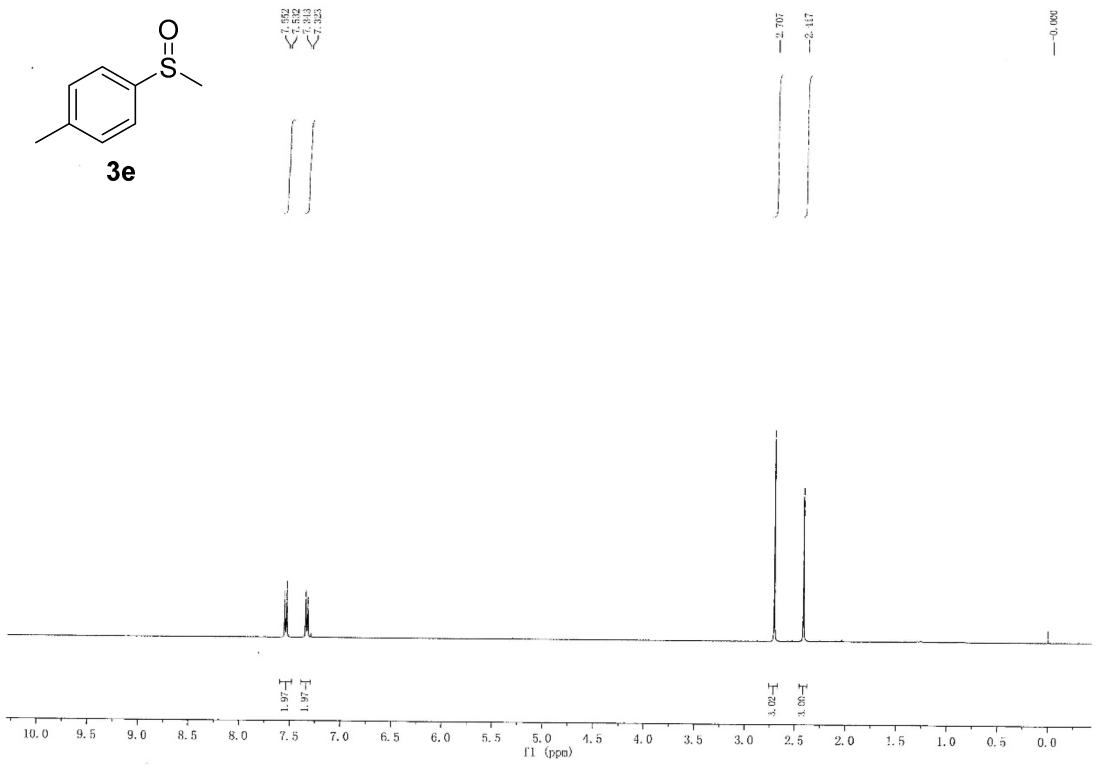
7.552
7.532
7.343
7.323
2.707
2.417
0.000
1.97
1.97
3.02
3.00
f1 (ppm)

3e

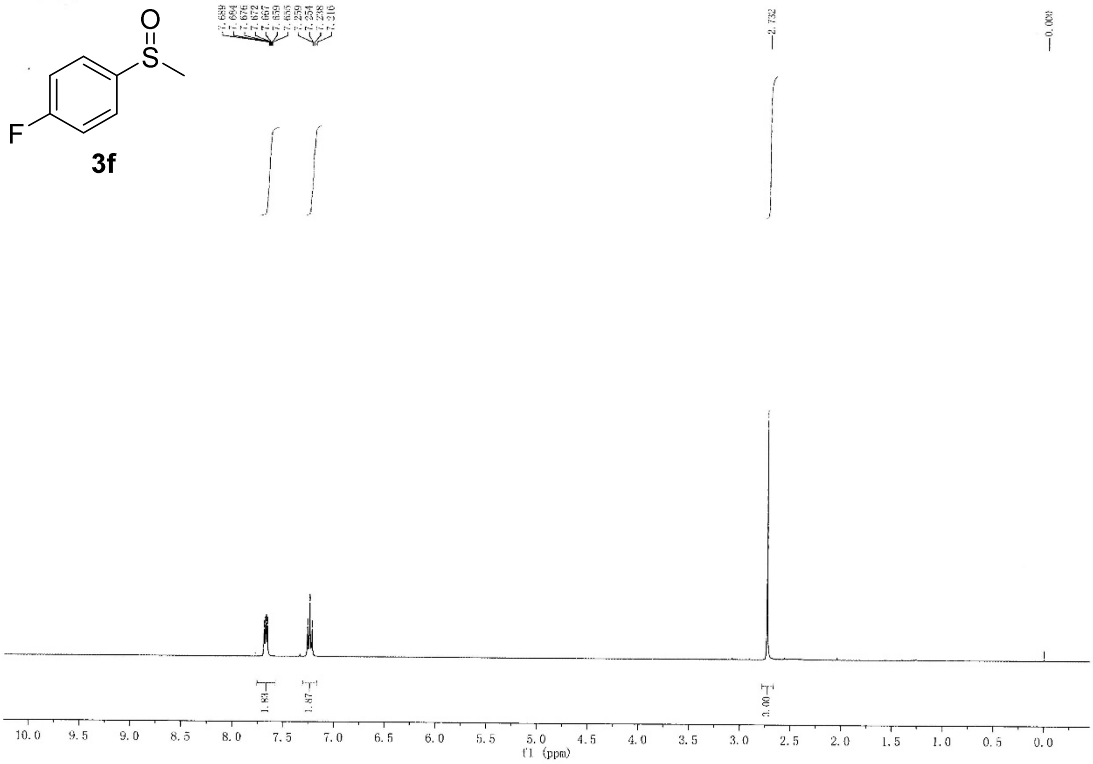
2.732
0.000
3.00
f1 (ppm)

3f

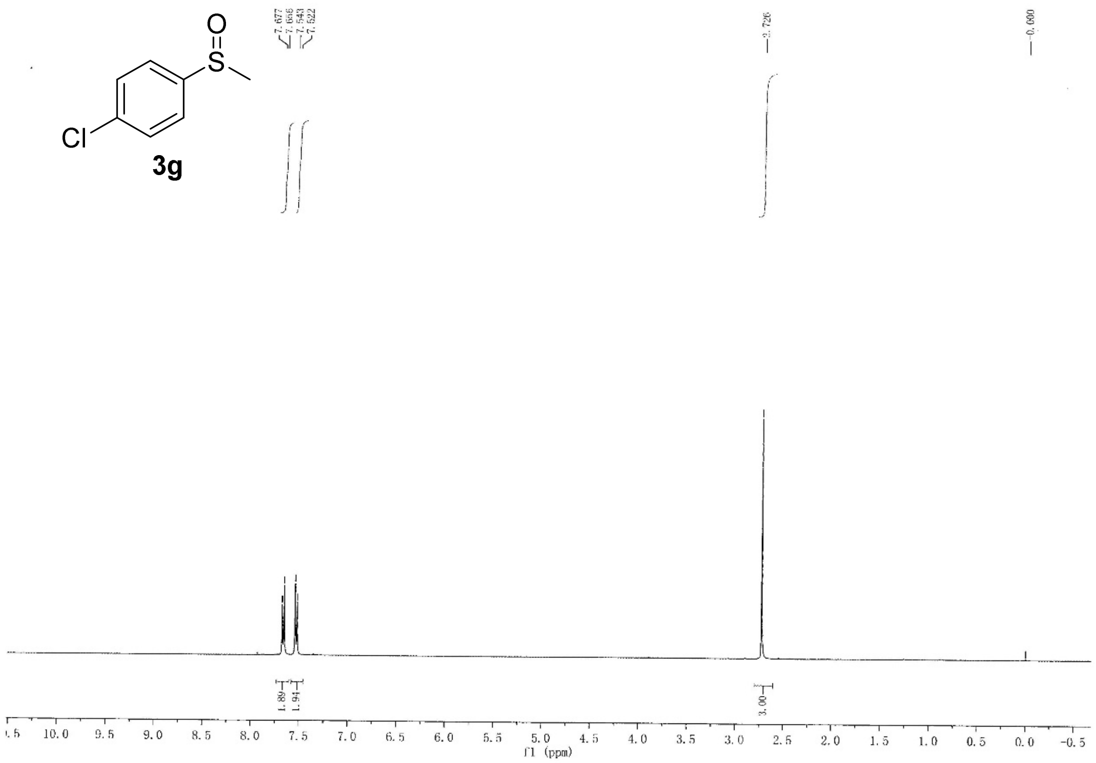
Cl
3g
7.677
7.656
7.543
7.522
2.726
0.000
1.89
1.94
3.00
f1 (ppm)

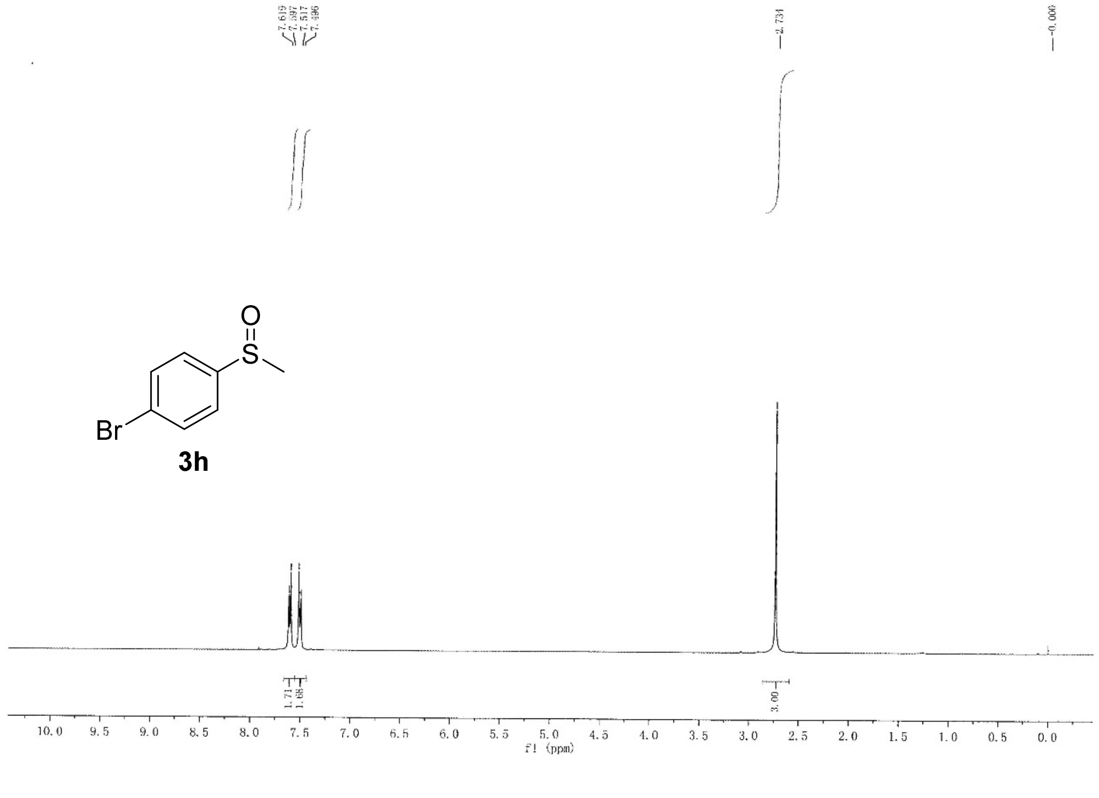
7.619
7.597
7.517
7.496
2.731
0.000
Br
3h
1.71
1.68
3.00
f1 (ppm)

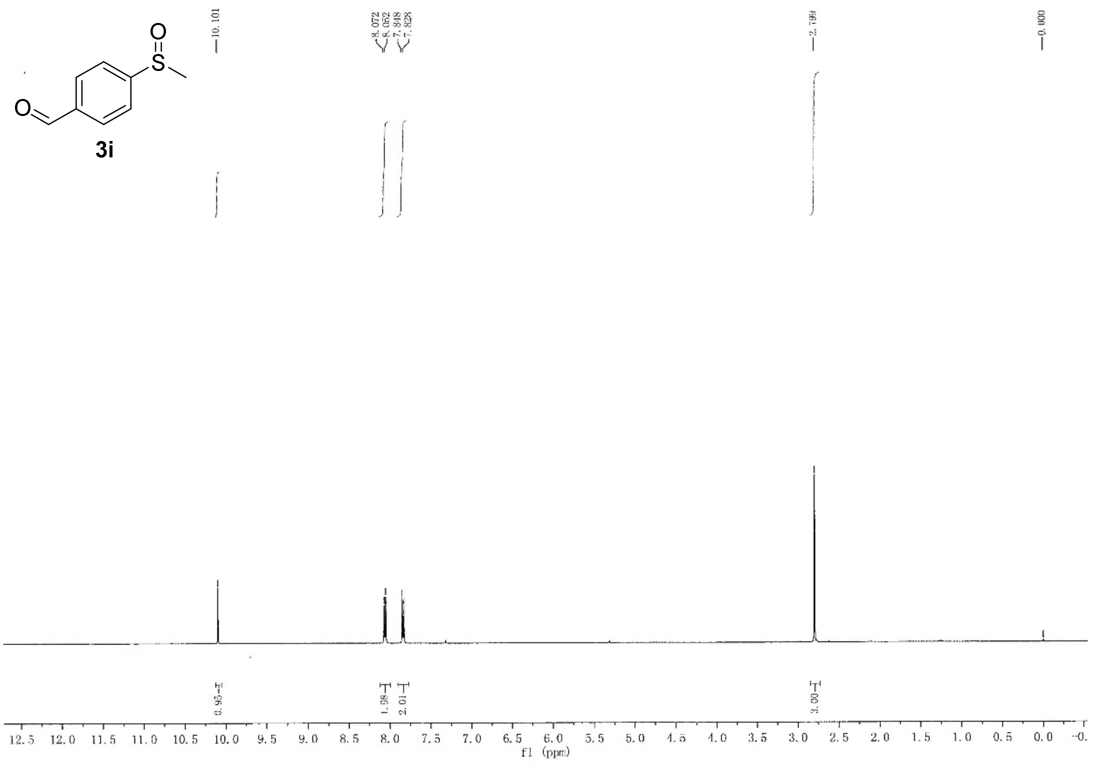
3i
10.101
8.072
8.052
7.848
7.828
2.799
0.000
0.95
1.98
2.01
3.00
f1 (ppm)

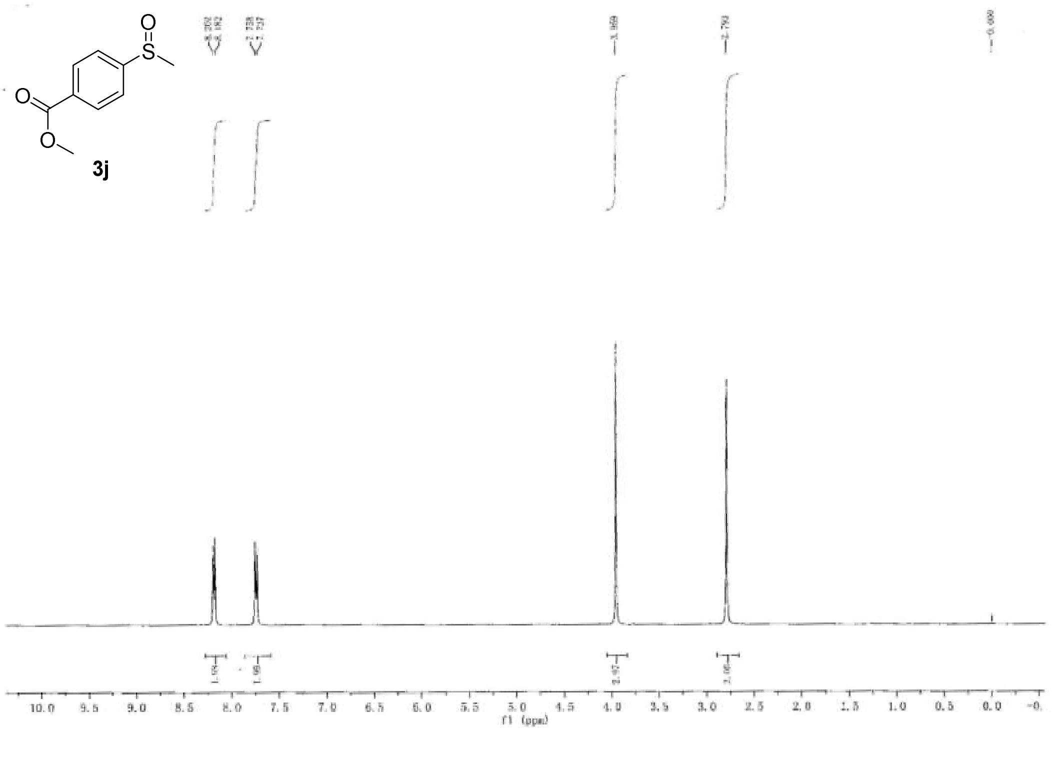
3j
3.959
2.793
f1 (ppm)

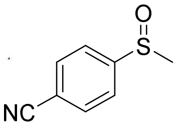

3k

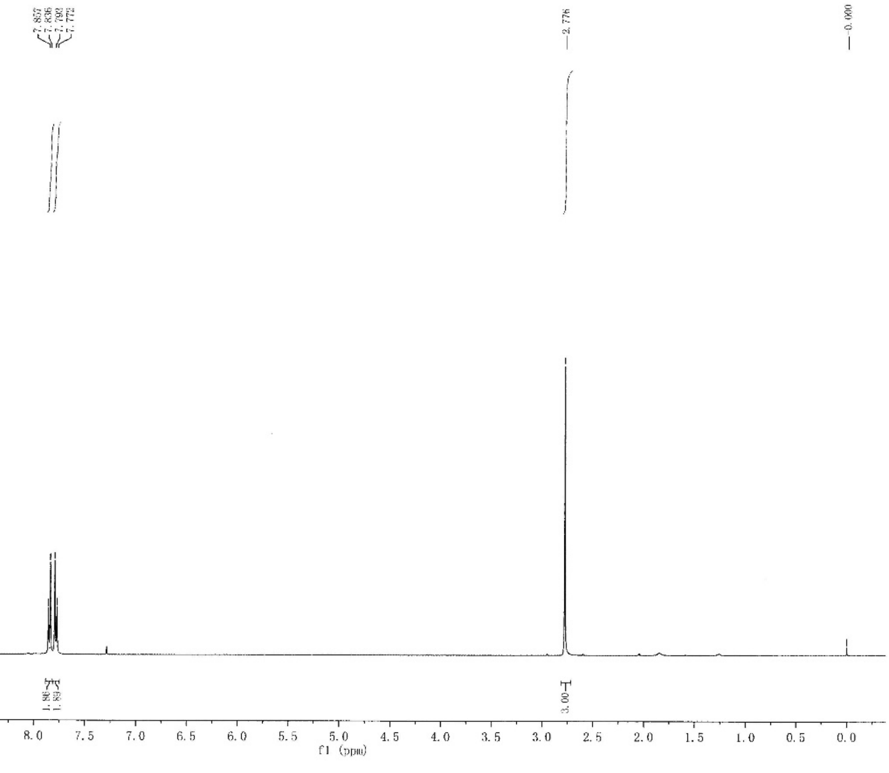

7.857
7.836
7.792
7.772
2.776
0.000
3.00
10.0 9.5 9.0 8.5 8.0 7.5 7.0 6.5 6.0 5.5 5.0 4.5 4.0 3.5 3.0 2.5 2.0 1.5 1.0 0.5 0.0
f1 (ppm)

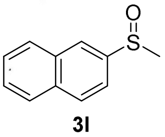

3l

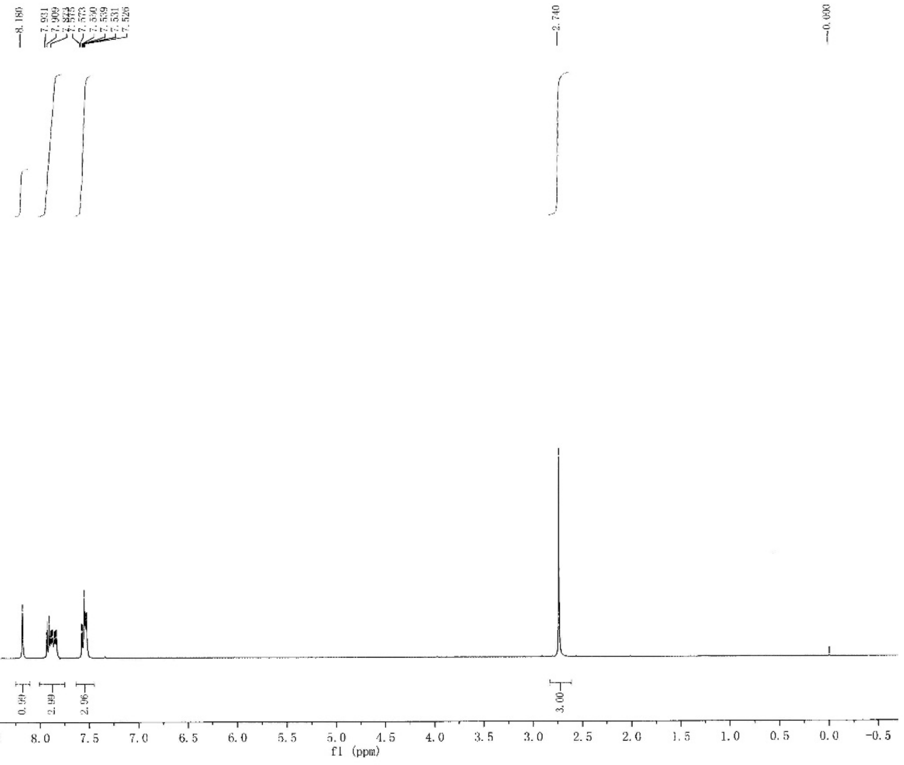

8.180
7.931
7.909
7.573
2.740
0.000
0.99
2.99
2.96
3.00
10.0 9.5 9.0 8.5 8.0 7.5 7.0 6.5 6.0 5.5 5.0 4.5 4.0 3.5 3.0 2.5 2.0 1.5 1.0 0.5 0.0 -0.5
f1 (ppm)

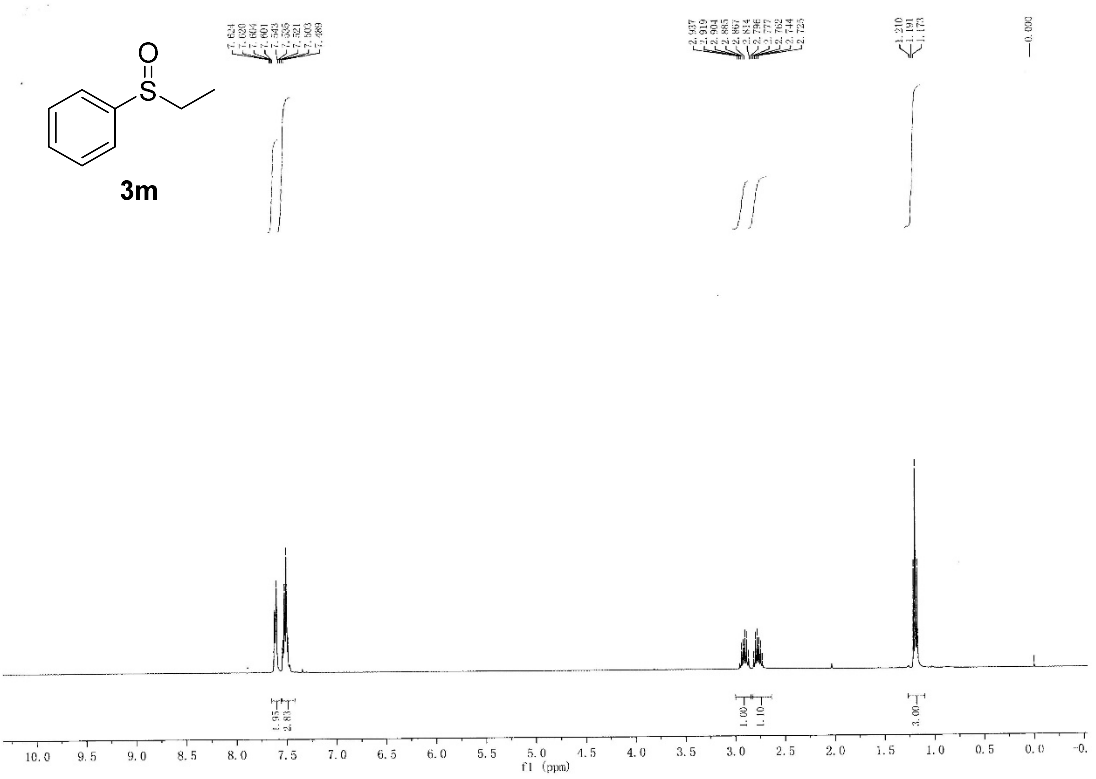
3m
f1 (ppm)

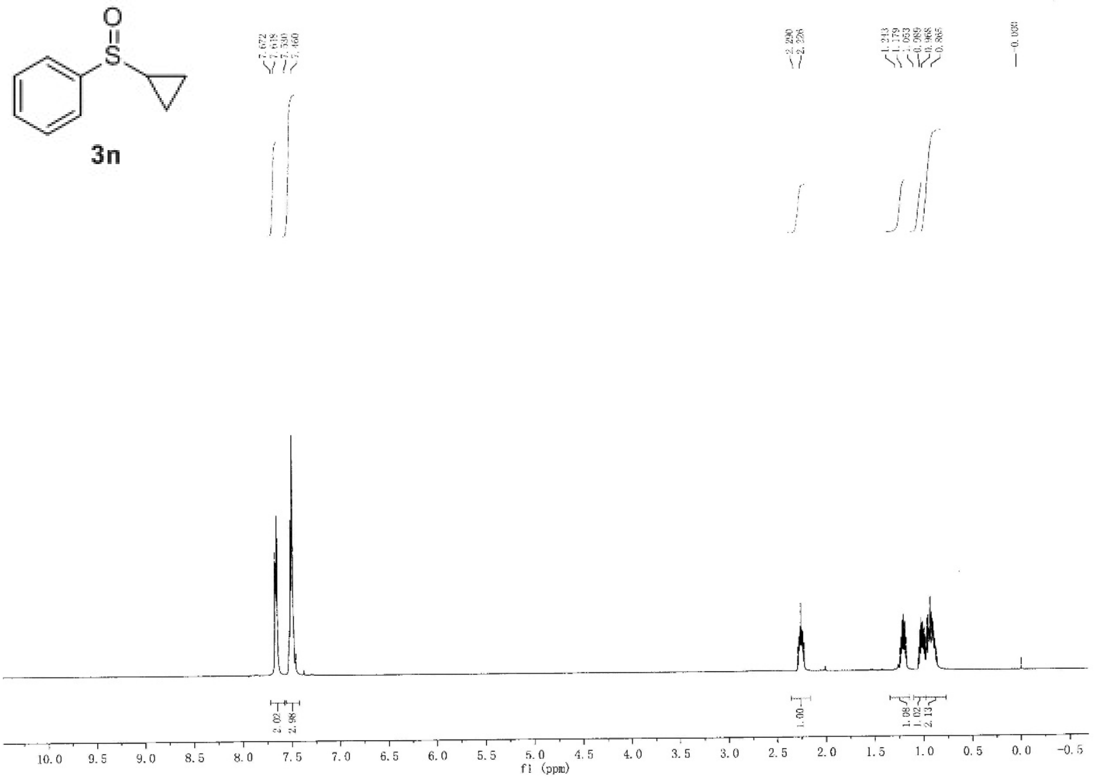
3n
f1 (ppm)

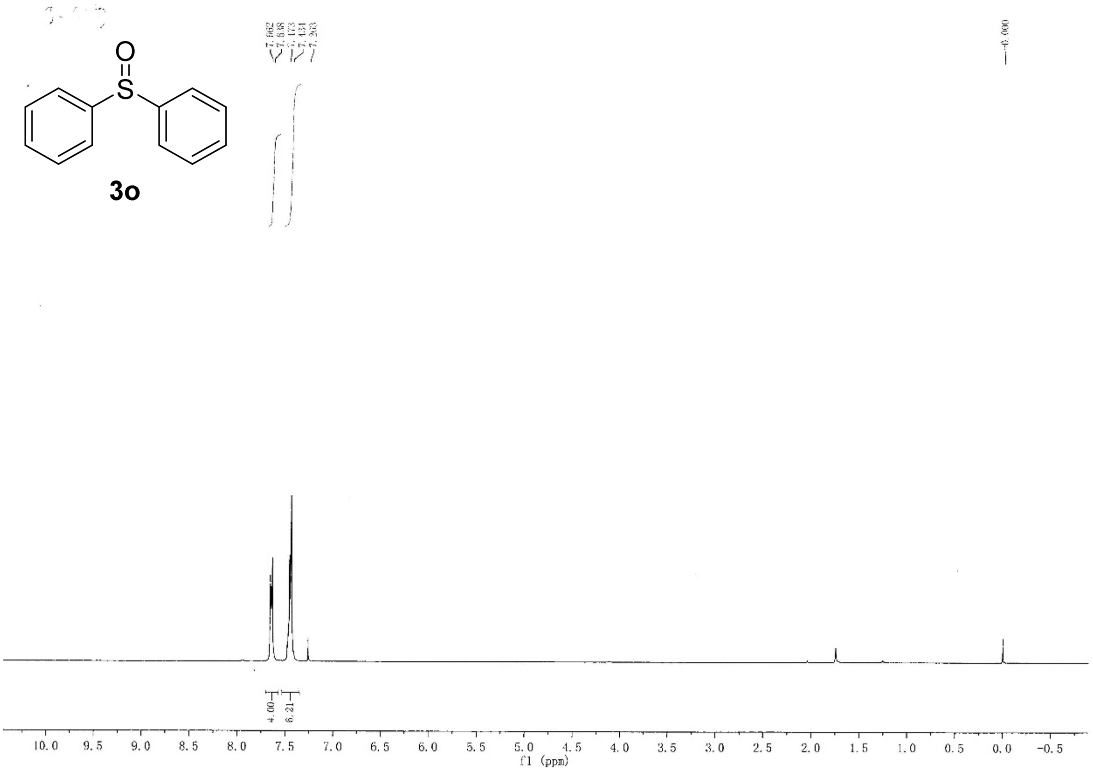

3o

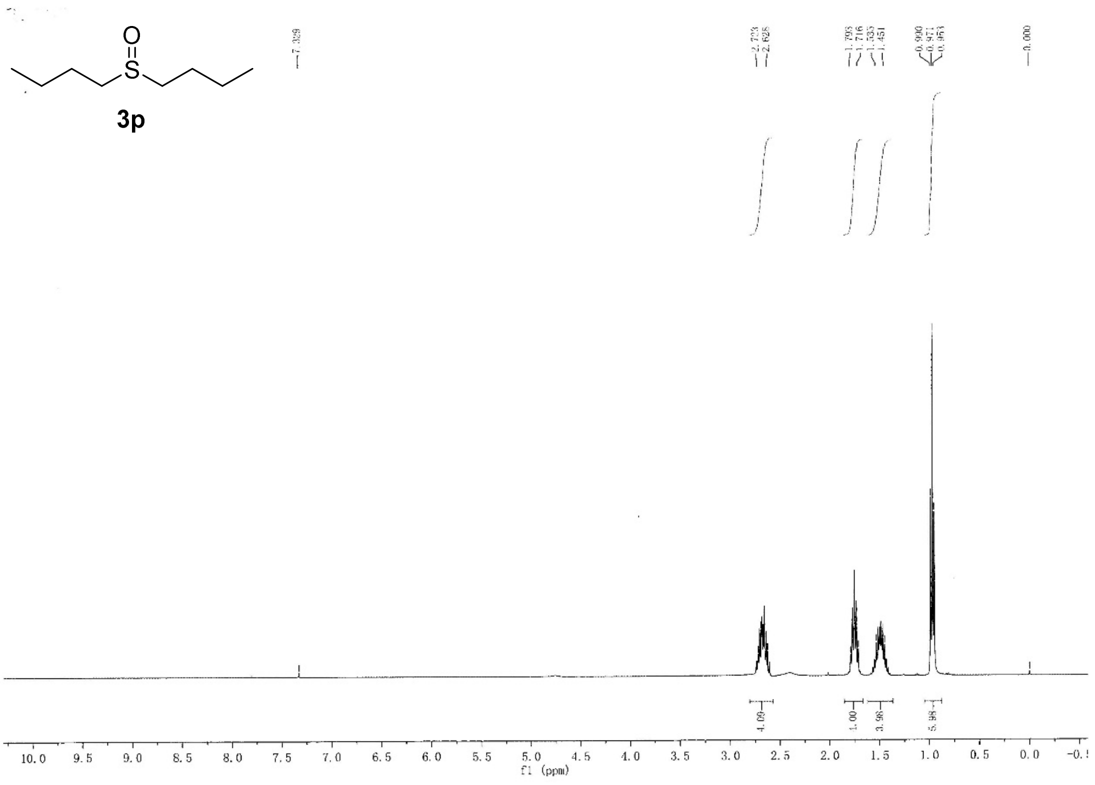

3p

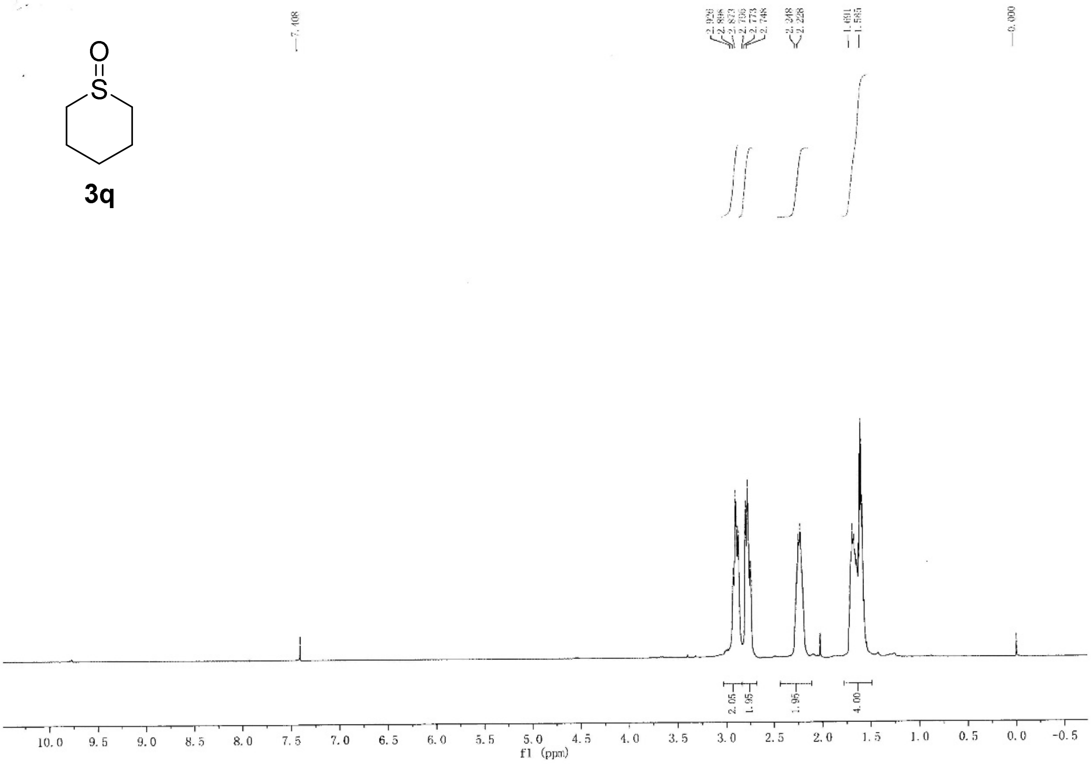

3q

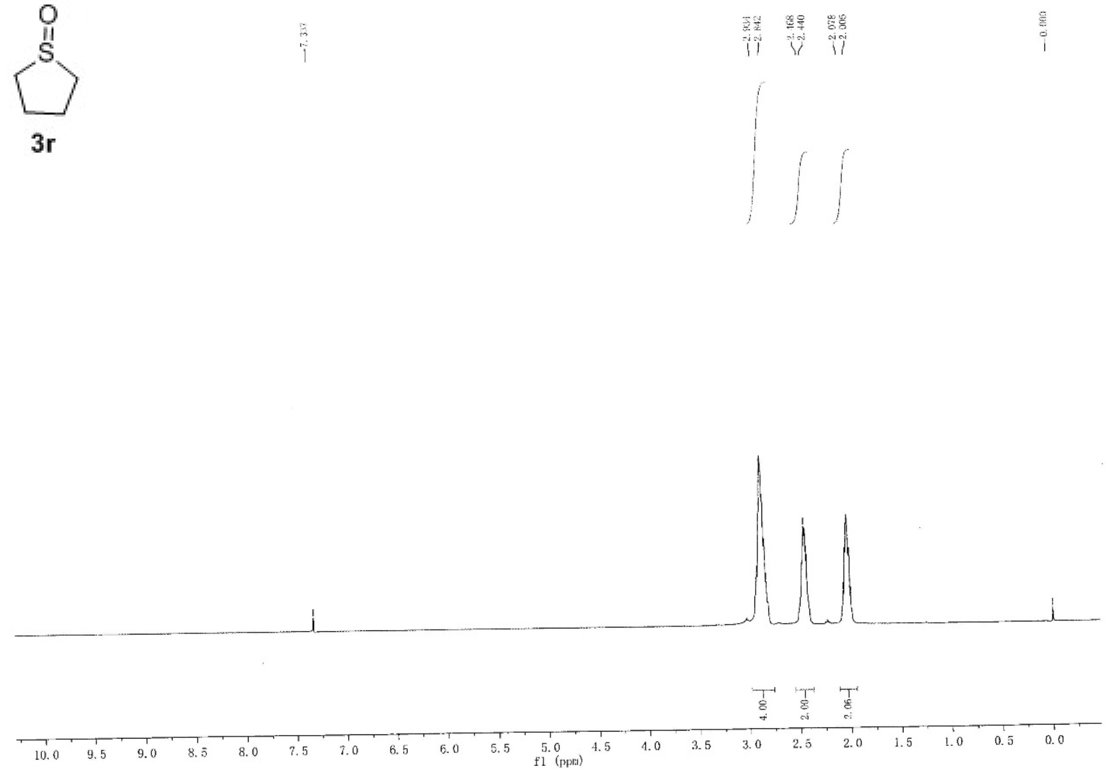

3r

References

1. Safraz, K., Panlo, L. B., Vladimir, A. K., Edwin, M. S. & Mikhail, S. S. Synthesisi of s-pixyl derivatives for mass spectrometric applications. *Synlett*. **16**, 2453-2456 (2005).
2. Hashem, S. & Ali, R. A novel and efficient method for the synthesis of new hydroxythioxanthone derivatives. *Synthesis.* **17**, 2900-2904 (2004).
3. Yuta, U., Takashi, N. & Takamitsu, H. Rhodium-catalyzed ipso-borylation of alkylthioarenes via C-S bond cleavage. *Org. Lett.* **18**, 2758-2761 (2016).
4. Keigo, K., Tomohisa, H. & Noritaka, M. Highly efficient oxidation of sulfides with hydrogen peroxide catalyzed by $[SeO_4\{WO(O_2)_2\}_2]^{2-}$. *Chem. Commun*. 3958-3960 (2005).
5. Hendriks, C., Lamers, P., Engel, J. & Bolm, C. Sulfoxide-to-sulfilimine conversions: use of modified burgesstype reagents. *Adv. Syth. Catal.* **355**, 3363-3368 (2016).
6. Zhao, L. L., Zhang, H. Y. & Wang, Y. H. Dirhodium(II)-catalyzed sulfide oxygenations: catalyst removal by coprecipitation with sulfoxides. *J. Org. Chem.* **81**, 129-136 (2016).
7. Fernandez, S., Eberhart, A. J. & Procter, D. J. Metal-free CH-CH-type cross-coupling of arenes and alkynes directed by a multifunctional sulfoxide group. *J. Am. Chem. Soc.* **138**, 790-793 (2016).
8. Pandya, V. et al. Synthesis and structure-activity relationship of potent, selective and orally active anthranilamide-based factor Xa inhibitors: Application of weakiy basic sulfoximine group as novel S4 binding element. *Eur. J. Med. Chem*. **58**, 136-152 (2012).
9. Gu, X. Y. et al. A simple metal-free caralyytic sulfoxidation under visible light and air. *Green Chem*. **15**, 357-361 (2013).
10. Yang, C. B., Jin, Q. P., Zhang, H., Liao, J., Zhu, J., Yu, B. & Deng, J. G. Tetra-(tetraalkylammonium)octamolybdate catalysts for selective oxidation of sulfides to sulfoxides with hydrogen peroxide. *Green Chem*. **11**, 1401-1405 (2009).
